# Mediterranean Dietary Pattern Adjusted for CKD Patients: The MedRen Diet

**DOI:** 10.3390/nu15051256

**Published:** 2023-03-02

**Authors:** Claudia D’Alessandro, Domenico Giannese, Vincenzo Panichi, Adamasco Cupisti

**Affiliations:** Department of Clinical and Experimental Medicine, University of Pisa, Via Roma 67, 56126 Pisa, Italy; claudia.dalessandro@unipi.it (C.D.); domenico.giannese@phd.unipi.it (D.G.); vincenzo.panichi@unipi.it (V.P.)

**Keywords:** Mediterranean diet, chronic kidney disease, CKD stage 3, dietary treatment, renal diet

## Abstract

A number of studies in the general population showed that healthy dietary patterns, such as the Mediterranean Diet, can improve or prevent the development of several chronic diseases and are associated with a significant reduction in all-cause and cardiovascular mortality. The Mediterranean diet may also have favorable effects for the prevention of chronic kidney disease (CKD), but no evidence of renoprotection exists in CKD patients. The Mediterranean Renal (MedRen) diet is an adaptation of the Mediterranean diet recommendations comprising a quantitative reduction in the RDA values of protein, salt and phosphate intake for the general population. Hence, MedRen supplies 0.8 g/Kg of protein, 6 g of salt and less than 800 mg of phosphate daily. Obviously, there is a preference for products of plant origin, which contain more alkali, fibers, unsaturated fatty acids than animal-based food. The MedRen diet can be implemented easily in mild-to-moderate stages of CKD with good results, both in terms of adherence to prescriptions and metabolic compensation. In our opinion, it should be the first step of CKD stage 3 nutritional management. This paper describes the features and reports our experience in the implementation of the MedRen diet as an early nutritional approach to CKD.

## 1. Introduction

Chronic kidney disease (CKD) is a widespread and progressive degenerative condition that leads to end-stage kidney disease (ESKD) requiring dialysis or transplantation. CKD is a quite complex and multifaced disease, and its care management also involves partners and family members, negatively affecting their quality of life. The prevalence of CKD, approximately 10% of the general population, is growing due to the increase in life expectancy and of the metabolic abnormalities typical of the Western countries, namely, overweight, obesity, diabetes, dyslipidemia and arterial hypertension. It has been predicted that CKD will rise in the sad ranking of the main causes of death from 16th place in 2016 to 5th place in 2040 [1].

The association between medications and dietary treatment can guarantee good metabolic control, the prevention of the signs and complications related to the loss of kidney function, and a delay to the start of dialysis [2]. The leitmotiv of the nutritional therapy is the control of sodium, phosphorus and protein intake, within a diet predominantly composed of plant-origin food [3]. These aspects are characterized as healthy dietary patterns—dietary approaches to stop hypertension (DASH) or Mediterranean diets [4,5]. In addition, it is well known that dietary salt restriction is effective for lowering proteinuria and arterial blood pressure in order to limit progressive kidney damage [6,7,8,9]. In particular, Slagman et al., in a multicenter crossover randomized controlled trial involving patients with nondiabetic nephropathy, showed that moderate dietary sodium restriction alongside angiotensin-converting enzyme inhibition was more effective for lowering proteinuria and blood pressure than the dual blockade of the angiotensin receptor blocker on top of angiotensin-converting enzyme inhibitor, but on high sodium intake [6].

Similarly, Vogt et al. showed that the addition of a low-sodium diet in non-diabetic proteinuric patients treated with losartan reduced proteinuria by enhancing the antiproteinuric effect of the drug, contributing a further 25% reduction; combining a low-sodium diet with hydroclorothiazide, the effect on proteinuria was an overall reduction of 70% from baseline [7].

Moreover, Lambers Heerspink et al. showed a protective effect on renal and cardiovascular outcomes in patients with type 2 diabetic nephropathy treated with angiotensin receptor blockers following a low-sodium diet compared to patients with higher sodium intake [8].

The importance of sodium restriction in the management of patients with CKD to reduce cardiovascular risk and the risk for kidney disease progression was also emphasized by McMahon et al. in a double-blind placebo-controlled randomized crossover trial involving stage 3–4 CKD patients with high blood pressure. Salt restriction was shown to significantly reduce blood pressure and extracellular fluid volume and had a positive effect, i.e., a significant reduction of, albuminuria and proteinuria. Interestingly the extent of the change was greater in CKD patients rather than patients without kidney failure, suggesting that patients with CKD are more sensitive to salt restriction [9].

Similarly, the control/restriction of protein intake is the mainstay of nutritional treatment of CKD, as well as a limitation of the effective phosphate load [3].

It is known that high protein intake (namely > 1.2 g of protein per kilogram of body weight) affects renal hemodynamics, increasing renal blood flow, intraglomerular pressure and glomerular filtration rate. These changes may threaten kidney health in the long term, leading to accelerated glomerulosclerosis. Conversely, protein restriction reduces intraglomerular pressure, renal workload, and has favorable metabolic effects that result in the prevention/correction of the signs and symptoms of chronic kidney insufficiency.

The limitation of phosphate intake, even in the early stages of CKD, is able to improve mineral metabolism, inducing a decrease in FGF-23 and PTH and an increase in calcitriol serum levels [3].

The aim of this article is to report our experience of implementing the early dietary management of CKD. We used a Mediterranean diet, including quantitative limitations of the sodium, protein and phosphate intake recommended to the general population. This approach has been named the Mediterranean Renal Diet.

## 2. The Mediterranean Diet, Going over Its History

Everything started in the 1950s when Ancel Keys (chair of Hygiene and Physiology at the University of Minnesota, Minneapolis, MN, USA) planned the “Seven Country Study” with the aim to evaluate the incidence of cardiovascular disease in Italy, Greece, Finland, Netherlands, ex-Yugoslavia, USA and Japan [10]. The study highlighted that the populations bordering the Mediterranean basin had a lower prevalence of cardiovascular diseases and a lower incidence of death, due to stroke or heart attack, compared to the others [10,11]. Further studies confirmed that dietary habits based on the lifestyle of these Mediterranean countries could be useful in the prevention of several metabolic diseases, namely, cardiovascular diseases, hypertension, diabetes, dyslipidemia, obesity, metabolic syndrome, nonalcoholic steatohepatitis, cancer, etc. [12,13,14,15].

The Mediterranean diet is also characterized by the presence of “nutraceutical” substances or molecules with proven beneficial and protective effects [16]. Nutraceuticals are normally derived from plants, foods, and microbial sources. Examples of nutraceuticals present in foods of the Mediterranean Diet are prebiotics (galacto-oligosaccharides, inulin), antioxidants (resveratrol, capsaicin), polyunsaturated fatty acids (omega-3), an adequate omega-6/omega-3 fatty acid ratio and vitamins (vitamin C, vitamin E). It is claimed that these substances can prevent chronic diseases, promote health, delay aging and increase life expectancy [12,17,18].

The Mediterranean diet has a solid background of peasant culture and tradition. The agricultural world had, and still has, vital importance for society. The food habits of ancient Greece and the Roman Empire were essentially based on the “Bread–Oil–Wine” triad together with sheep and goat cheeses, fruit and vegetables, legumes, a small amount of meat and preference for fish and seafood. The Mediterranean diet is, therefore, based on whole bread, olive oil and red wine on top of vegetables, fresh and dried fruit, proteins of both vegetable (legumes) and animal origin, especially fish (sardines, anchovies, spatulas, etc.), milk and dairy products, white meat (chicken, rabbit, turkey, etc.). Refined sugars, animal fats and red meat are not part of a Mediterranean diet (Table 1) [19,20]. In the last few years, the Mediterranean diet has also been re-evaluated for its beneficial effects in terms of sustainability for its low environmental impact, biodiversity and favorable local economic returns [17].

**Figure 1:**
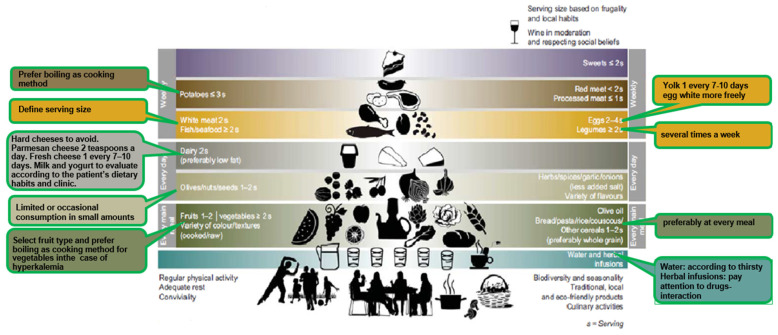
The new Mediterranean Diet Pyramid, with the addition of boxes (with green outline) on the right and the left side of the pyramid, reporting the main changes to adapt the Mediterranean diet to CKD, leading to the Mediterranean Renal Diet. Table 1 shows more details. Modified from Bach-Faig A et al. [21].

Nutrition is a physiological and health issue, but eating is more than having food to survive: it is a cultural and social phenomenon. Considering these aspects, the Mediterranean Diet Foundation, together with the Forum on Mediterranean Food Cultures, developed a consensus position on a new revised Mediterranean diet representation pyramid (Figure 1) [21,22]. The new revised Mediterranean diet and food lifestyle pyramid not only provides recommendations regarding the proportion and frequency of food consumption, but also includes cultural and lifestyle suggestions. Although these elements are represented outside of the pyramid, they are placed at its base, and this is the innovative aspect of this educational tool. The adoption of a healthy lifestyle and the preservation of cultural elements are considered mandatory to obtain the benefits of the Mediterranean diet and to preserve this cultural heritage [21,22].

## 3. Mediterranean Diet and Kidney Disease

The main recommendations of the Mediterranean diet in part meet the needs of the subject with CKD, such as a reduction in meat consumption, contributing to reduced animal protein intake; the use of plant-based foods, increasing consumption of plant-based proteins; the presence of high amount of fibers, vitamins, alkali and polyphenols, contributing to gut microbiota homeostasis and preventing inflammation; the use of extra virgin olive oil, rich in energy deriving from fats of vegetable origin and rich in polyphenols [23,24]. Some concerns remain on the potential risk of hyperkalemia linked to the high prevalence of plant-based foods. However, foods of vegetable origin supply alkali and reduce net endogenous acid production, so they are useful for preventing/correcting CKD related metabolic acidosis. The presence of fibers also promotes proper intestinal function by preventing constipation which, in turn, can cause hyperkalemia. As a whole, plant-dominant diets have no hyperkalemic effects because they also have some protective effects in respect to the increase in potassium serum levels [25]. Finally, fibers are known to have a favorable effect on the composition of gut microbiota, since fiber alterations are associated with a worse prognosis of CKD. In the last decade, or even more, the gut microbiota has been identified as a non-traditional and modifiable risk factor for patients with kidney disease and a possible action target to reduce the risk of cardiovascular damage that play an important role also in the progression of renal disease. Dietary changes are known to affect gut microbiota metabolism and composition. Adequate manipulation of food intake in CKD may reduce the uremic toxins (i.e., p-cresyl sulfate, indoxyl sulfate, trimethylamine-N-Oxide, indole-3 Acetic Acid) typically produced by gut microbiota that potentially increased the risk of cardiovascular and kidney damage. Fiber intake, provided by plant-based foods, promotes positive microbiota composition and metabolism, lowering the levels of uremic toxins produced by intestinal bacteria—this, combined with the other features of the Mediterranean diet, contributes to a reduction in cardiovascular risk in CKD patients [26,27,28]. A recent investigation conducted in the frame of the CORDIOPREV study (a prospective, randomized, controlled trial, including 1002 patients with coronary heart disease), evaluated the efficacy of 5 years of consuming a Mediterranean diet rich in extra-virgin olive oil on kidney function. The intervention, compared to a low-fat diet rich in complex carbohydrates, was able to reduce the decline in eGFR in coronary heart disease patients with type 2 diabetes. Patients with a mild reduction in eGFR benefited more from the consumption of the Mediterranean diet in terms of preservation of kidney function with time [29].

Ajjarapu et al. summarized the results from observational studies investigating associations between dietary patterns and renal outcomes in the general population published over a 10-year period. The main renal outcome was eGFR < 60 mL/min/1.73 m^2^. The authors showed that adherence to the Dietary Approaches to Stop Hypertension (DASH) and Med diets were significantly associated with a reduced risk of CKD incidence most of the studies highlighted [30]. Similar results were found by Khatri et al. who observed that every 1-point increase in the MeDiet score (a questionnaire used to assess adherence to Mediterranean diet—greater score indicates better adherence to a Mediterranean diet) was associated with decreased odds of incidence, eGFR < 60 mL/min per 1.73 m^2^, and decreased odds of being in the upper quartile of eGFR decline in a multiethnic cohort [31].

Asghari et al. studied the association between the Mediterranean diet score and the 6-year incidence rate of CKD in 1212 adults aged 30–71 years in the framework of the Tehran Lipid and Glucose Study. They saw a significant inverse association between the Mediterranean diet score and the risk of CKD incidents, indicating that stricter adherence to the Mediterranean diet had more favorable effects on the prevention of kidney disease [32].

Huang et al. showed an independent relationship between adherence to a Mediterranean diet and kidney function in a population-based cohort of 1110 elderly men. Greater adherence to Mediterranean diet was protective for kidney function and able to improve clinical outcomes in CKD patients [33]. Chrysohoou C et al. studied the association between adherence to the Mediterranean diet and markers of kidney function among more than 3000 healthy people with CKD. They found that greater adherence to the Mediterranean diet was independently associated with lower urea and creatinine serum levels and increased creatinine clearance [34]. Chauveau et al. examined the main recommendations of the Mediterranean diet and evaluated their suitability for patients with CKD and the pros and cons of the preponderant presence of vegetables [35]. As a whole, healthy dietary patterns significantly reduced the incidence of CKD and albuminuria in the general population [36]. A metanalysis of seven studies including 15,285 participants (follow up of 4 to 13 years) showed that subjects with CKD following a dietary pattern rich in vegetables, whole grains and cereals, fruit, fish, legumes, nuts, high in fiber and low in red meat, salt and refined sugars was consistently associated with a lower risk of death. Unfortunately, following a healthy dietary pattern was not significantly associated with a reduction in the risk of end-stage renal disease. The risk of ESRD among people with CKD was 1.04 (95% CI, 0.68 to 1.40).

Kelly et al. [37] states that healthy dietary patterns do not seem to exert sufficient protective power to protect the kidney from progressive damage in patients already suffering from CKD; hence, a more careful selection and use of foods must be implemented to obtain an even greater benefit.

## 4. The Mediterranean Renal Diet

Our personal experience confirms that a dietary intervention, based on the main recommendations of the Mediterranean diet, may also have an application in mild-to-moderate stages of CKD, provided that some quantitative changes to the intake of nutrients are made to tailor the diet to the patient’s clinical needs. This so-called Mediterranean Renal (MedRen) diet is helpful in terms of adherence to dietary prescriptions, since it resembles a traditional dietary pattern and offers positive results in maintaining good metabolic status even in presence of moderate reduction in kidney function [38].

The main features of the MedRen include limitations of the amount of protein, sodium and phosphate intake, but high intake of fibers and olive oil, and foods of plant origin.

The MedRen diet meets the characteristics described for the plant-dominant (PLADO) diet particularly suitable for patients with CKD [39,40].

In our outpatient CKD clinic, patients with stage 3 CKD receive dietary counselling corresponding to the principles of the Mediterranean diet, but with quantitative prescriptions of nutrients based on the metabolic or clinical needs of the patient. The main features of this modified Mediterranean diet, here defined as MedRen, are shown in Figure 1 and reported with more detail in Table 1, third column.

The adaptations consist of more frequent consumption of plant-based protein, favoring the use of a combination of cereals and legumes several times a week instead of meat and fish and other sources of animal protein [41]. Plant foods, including legumes, have a lower acidifying effect partly due to the lower content of sulphurated amino acids. Moreover, fruits and vegetables are rich in organic salts whose metabolic pathway requires hydrogen ions contributing to an alkalizing effect Legumes are rich in phosphate, but phosphate of plant origin is present above all in the form of phytates; human beings do not have the phytase enzyme, so phosphate from plants is scarcely absorbable. Our patients received a recommendation regarding legume preparation, that is, to soak and boil them, and to discard the cooking water (rich in potassium and phosphate delivered by legumes during boiling procedure). Nuts are typically included in the Mediterranean diet but, in the case of CKD, they should be limited because of the high content of phosphate and potassium. Phosphate in nuts is obviously of plant origin and so less absorbable, but the total contents of phosphate and potassium are so high that they should be managed carefully and limited in amount, especially in those patients at risk of hyperphosphatemia or hyperkaliemia.

Establishing an adequate serving size for each patient is important, particularly regarding foods rich in proteins, salt and phosphorus. This does not mean “binding” patients to the use of kitchen scales but to educate them to understand what the right is serving to be able to better manage themselves even when they do not have meals at home. The risk of hyperkalemia is higher in the advanced stages, so the suggestions of dietary manipulation, e.g., using boiling as the preferred cooking method, is recommended [42,43,44]. The control of salt intake is mandatory and it is probably the top recommendation for patients with CKD, but this is part of the suggestion given by WHO for the general population.

Greater attention than that indicated by the standard Mediterranean diet should be paid to the consumption of milk and dairy products. This warning concerns hard cheeses to limit salt loading, whereas the restriction of fresh dairy is also necessary to control phosphorus intake considering that the alterations of the calcium–phosphorus metabolism occur very early during CKD [45,46].

Besides the quality of foods, a MedRen diet also involves defining the amount of some nutrients, namely, normalizing the intake of protein to 0.8 g/Kg/d, salt at 6 g daily and phosphate at 800–700 mg/d, are mandatory to obtain a diet that fits well with a moderate reduction in kidney function.

Moreover, the dietary management of CKD patients should not be related only to the residual renal function and its progression rate, but also depends on other aspects such as the socio-economic, psychological, and functional status of the patient—all aspects that play an important role in achieving and keeping a good level of adherence to the dietary prescriptions. In our experience, a stepwise intervention helps the patient to gradually approach a more suitable nutritional treatment for kidney disease [38,42]. In a previous case–control study, we evaluated the effects of pragmatic, patient-centered, stepwise nutritional support in the management of 823 patients with CKD on tertiary care. The nutritional intervention was offered to 305 patients to prevent or correct metabolic abnormalities and to maintain good nutritional status. It included the manipulation of sodium, phosphate, energy and protein dietary intakes. Dietary counselling was not implemented solely based on the CKD stage but also and above all on metabolic parameter (biochemical, urinary exams and nutritional parameter) and patients’ habits and needs [38]. As expected, the implementation of low-protein diets increased from stage 3b (10.2%) to stage 4 (60.2%) and stage 5 (91.4%). The Mediterranean Renal diet (at that time reported as a “Normal diet”) was prevalent in 41.5% of stage 3b and in 22.6% of stage 4 patients. Only 8% disliked the MedRen diet that was well accepted by 78% according to the results of a validated dietary satisfaction questionnaire [38].

Hence, the MedRen diet represents the shift from a healthy dietary pattern to a first-step renal diet, starting from the patient’s dietary habits and lifestyle. In this way, patients themselves “drive” the dietary counselling process, helping dietitians to implement a dietary recommendation that best suits the patients’ preferences and clinical needs.

The adoption of a healthy lifestyle and the preservation of cultural elements are considered mandatory to obtain all the benefits from the Mediterranean Diet and to preserve this cultural heritage. These aspects have a crucial role also in a CKD setting and must be maintained in the MedRen diet to have more chance to guarantee a good metabolic status and ensure good long-lasting concordance and adherence.

By several years, we have been implementing the MedRen diet in our CKD nutrition clinic. We report some personal data from a cohort of 93 patients (aged 64 ± 10 yrs) affected by Stage 3 CKD who underwent MedRen diet by 6 months at least, in stable clinical conditions and on standard pharmacological therapy. Urea and phosphate serum levels were compared with those of a historical cohort of unselected 223 patients (aged 66 ± 10 years) affected by stage 3 CKD matched for residual kidney function but with no nutritional treatment [47]. The patients on MedRen diet group had the same creatinine serum levels (1.63 ± 0.28 vs. 1.65 ± 0.34 mg/dL) and the same eGFR (42.7 ± 7.0 vs. 42.7 ± 7.7 mL/min*1.73 m^2^) when compared to the group of patients without any nutritional care; the BMI results were also very similar (28.8 ± 4.6 vs. 28.8 ± 4.8 Kg/m^2^). Additionally, urea serum levels were lower in the MedRen group (60.7 ± 17.6 vs. 70.4 ± 23.1 mg/dL, *p* < 0.001), suggesting a lower effective protein intake. Similarly, phosphorus (3.1 ± 0.5 vs. 3.6 ± 0.6 mg/dL, *p* < 0.001) and PTH (71 ± 44 vs. 102 ± 83 pg/mL, *p* < 0.01) serum levels were lower in the MedRen group, suggesting an amelioration of CKD–MBD, as expected by the control of protein and phosphate intake. These are only personal data that need to be confirmed in larger series and controlled trials.

## 5. Conclusions

The application of healthy dietary patterns, as the Mediterranean diet, may exert favorable effects for the prevention of CKD and cardiovascular mortality. In patients with overt CKD, healthy dietary patterns are associated with reduced mortality risk, but are not able to prevent the progressive loss of residual kidney function.

The Mediterranean Renal diet represents an adjustment of the Med diet recommendations, including quantitative restriction of protein, salt and phosphate, so that it can be implemented in mild-to-moderate stages of CKD. The MedRen, consisting of dietary prescriptions and healthy lifestyle suggestions, helps the patient to gradually approach a more suitable nutritional treatment for CKD, and represents a gentle shift to a low-protein regimen, if needed [48].

The prescription of a low-protein (0.6 g/kg/day) diet or very low-protein (0.3–0.4 g/kg/d) diet supplemented with essential amino acids and keto acids is a further dietetic intervention suitable in the advanced stages of CKD when less restrictive diets achieved unsatisfactory results [49,50].

## Figures and Tables

**Table 1:** Main characteristics of the Mediterranean diet (2nd column) and the Mediterranean Renal diet (3rd column). The third column listed changes to adapt Med diet to CKD patients’ needs.

	Mediterranean Diet	Mediterranean Renal Diet
Cereals (bread, wheat, corn, pasta, bread, rice, barley, etc.).	1–2 times a day (preferably whole grain)	Daily use—should be consumed at every meal
Olive oil	Every meal	Rich in oleic acid with antioxidant properties. Important source of vegetable fats. Animal fats, like butter or cream, should only be allowed in patients with poor appetite, to increase energy density and to improve food palatability
Dairy	2 times a day (preferably low fat)	Source of animal protein but also rich in phosphate and salt (hard cheeses in particular). Prefer fresh cheese once every 7–10 days and limit/avoid hard cheese. As regards milk and yogurt, dietitians will evaluate their introduction on the basis of patient’s dietary habits and clinic. Consider replacing milk with plant-based drink such as rice, oats or almond drink. Parmesan cheese is a hard cheese rich in phosphorus and salt, so just small amounts over pasta should be allowed
Nuts/seeds	1–2 times a day	Rich in potassium and phosphorus: limited or occasional consumption
Red meat White meat	<2 times a week 2 times a week	Protein content of “white” or “red” meat is more or less the same. Pay attention to the serving defined by the dietician and limit its consumption to 1–2 times a week
Processed meat	≤Once a week	Processed meat (sausages, cold cuts) is rich in salt and may potentially contain phosphorus-based preservatives; for these reasons, its consumption should not be recommended
Fish and seafood	≥2 a week	Fish is preferable to meat for the good quality of fats but the serving size should be respected to avoid exceeding protein intake. Fish and seafood can be consumed 2–3 times a week
Eggs	2–4 a week	Yolk is rich in phosphorus; thus, the occasional consumption of whole egg should be considered. Conversely, egg white has very little phosphorus and a large amount of proteins of high biologicalvalue, so more frequent consumption could be suggested
Legumes (beans, chickpeas, peas, lentils, etc.).	≥2 a week	Consumption several times a week should be suggested. Legumes contain protein of good biological value and should be used as a substitute for meat, fish, etc., together with cereals to guarantee the introduction of all essential amino acids. Recommendations are given regarding their preparation, that is to use legumes after boiling and discarding cooking water.
Fruits Vegetables	1–2 a day ≥2 a day	Fruits and vegetables are rich in vitamins, fiber and minerals. Given the high potassium content their consumption may require some precautions (e.g., using boiling as cooking method)

## Data Availability

Not applicable.
